# Engaging Staff, Family, and Older Adults Living With Dementia in Nursing Home Research

**DOI:** 10.1093/geroni/igaf122.987

**Published:** 2025-12-31

**Authors:** Andreina Jimenez

**Affiliations:** New York University, New York, New York, United States

## Abstract

Despite the importance of inclusive research, conducting studies in nursing home communities with Black, Latino, and White older adults living with dementia, family care partners, and nursing home staff poses unique research engagement challenges such as recruitment, consent, and implementation of study activities. The significant strain the COVID-19 pandemic placed on the nursing home workforce has further hindered research engagement efforts. This presentation will discuss the obstacles encountered and strategies implemented in research engagement activities within our ongoing research with Black, Latino, and White older adults living with dementia, family care partners, and nursing home staff. It will emphasize the importance of collating traditional recruitment tactics (i.e. email, phone calls, and snowball methods) with a more modern approach using text, referral bonuses, and social media. Leveraging findings from 12 nursing home interactions, we will highlight the importance of transparency, culturally sensitive approaches, and trust-building strategies in overcoming research engagement challenges with nursing home executive leadership and nursing home research participants. These insights are important for informing future recruitment strategies, research methodologies, and approaches.

